# The Genetic Transformation of Chlamydia pneumoniae

**DOI:** 10.1128/mSphere.00412-18

**Published:** 2018-10-10

**Authors:** Kensuke Shima, Maximilian Wanker, Rachel J. Skilton, Lesley T. Cutcliffe, Christiane Schnee, Thomas A. Kohl, Stefan Niemann, Javier Geijo, Matthias Klinger, Peter Timms, Thomas Rattei, Konrad Sachse, Ian N. Clarke, Jan Rupp

**Affiliations:** a Department of Infectious Diseases and Microbiology, University of Luebeck, Luebeck, Germany; b German Center for Infection Research (DZIF), Partner Site, Hamburg-Luebeck-Borstel-Riems, Germany; c Molecular Microbiology Group, Faculty of Medicine, University of Southampton, Southampton General Hospital, Southampton, United Kingdom; d Institute of Molecular Pathogenesis, Friedrich-Loeffler-lnstitute (Federal Research Institute for Animal Health), Jena, Germany; e Molecular and Experimental Mycobacteriology, Research Center Borstel, Borstel, Germany; f Division of Computational Systems Biology, University Vienna, Vienna, Austria; g Institute of Anatomy, University of Luebeck, Luebeck, Germany; h University of Sunshine Coast, Maroochydore, Australia; i RNA Bioinformatics and High-Throughput Analysis, Faculty of Mathematics and Computer Science, Friedrich-Schiller-Universität Jena, Jena, Germany; University of Iowa

**Keywords:** *Chlamydia felis*, *Chlamydia pneumoniae*, genetic manipulation, plasmid shuttle vector, plasmid tropism, transformation

## Abstract

We demonstrate the genetic transformation of Chlamydia pneumoniae using a plasmid shuttle vector system which generates stable transformants. The equine C. pneumoniae N16 isolate harbors the 7.5-kb plasmid pCpnE1. We constructed the plasmid vector pRSGFPCAT-Cpn containing a pCpnE1 backbone, plus the red-shifted green fluorescent protein (RSGFP), as well as the chloramphenicol acetyltransferase (CAT) gene used for the selection of plasmid shuttle vector-bearing C. pneumoniae transformants. Using the pRSGFPCAT-Cpn plasmid construct, expression of RSGFP in koala isolate C. pneumoniae LPCoLN was demonstrated. Furthermore, we discovered that the human cardiovascular isolate C. pneumoniae CV-6 and the human community-acquired pneumonia-associated C. pneumoniae IOL-207 could also be transformed with pRSGFPCAT-Cpn. In previous studies, it was shown that *Chlamydia* spp. cannot be transformed when the plasmid shuttle vector is constructed from a different plasmid backbone to the homologous species. Accordingly, we confirmed that pRSGFPCAT-Cpn could not cross the species barrier in plasmid-bearing and plasmid-free C. trachomatis, C. muridarum, C. caviae, C. pecorum, and C. abortus. However, contrary to our expectation, pRSGFPCAT-Cpn did transform C. felis. Furthermore, pRSGFPCAT-Cpn did not recombine with the wild-type plasmid of C. felis. Taken together, we provide for the first time an easy-to-handle transformation protocol for C. pneumoniae that results in stable transformants. In addition, the vector can cross the species barrier to C. felis, indicating the potential of horizontal pathogenic gene transfer via a plasmid.

**IMPORTANCE** The absence of tools for the genetic manipulation of C. pneumoniae has hampered research into all aspects of its biology. In this study, we established a novel reproducible method for C. pneumoniae transformation based on a plasmid shuttle vector system. We constructed a C. pneumoniae plasmid backbone shuttle vector, pRSGFPCAT-Cpn. The construct expresses the red-shifted green fluorescent protein (RSGFP) fused to chloramphenicol acetyltransferase in C. pneumoniae. C. pneumoniae transformants stably retained pRSGFPCAT-Cpn and expressed RSGFP in epithelial cells, even in the absence of chloramphenicol. The successful transformation in C. pneumoniae using pRSGFPCAT-Cpn will advance the field of chlamydial genetics and is a promising new approach to investigate gene functions in C. pneumoniae biology. In addition, we demonstrated that pRSGFPCAT-Cpn overcame the plasmid species barrier without the need for recombination with an endogenous plasmid, indicating the potential probability of horizontal chlamydial pathogenic gene transfer by plasmids between chlamydial species.

## Abstract (precis)

The absence of tools for the genetic manipulation of C. pneumoniae has hampered research into all aspects of its biology. In this study, we established a novel reproducible method for C. pneumoniae transformation based on a plasmid shuttle vector system. We constructed a C. pneumoniae plasmid backbone shuttle vector, pRSGFPCAT-Cpn. The construct expresses the red-shifted green fluorescent protein (RSGFP) fused to chloramphenicol acetyltransferase in C. pneumoniae. C. pneumoniae transformants stably retained pRSGFPCAT-Cpn and expressed RSGFP in epithelial cells, even in the absence of chloramphenicol. The successful transformation in C. pneumoniae using pRSGFPCAT-Cpn will advance the field of chlamydial genetics and is a promising new approach to investigate gene functions in C. pneumoniae biology. In addition, we demonstrated that pRSGFPCAT-Cpn overcame the plasmid species barrier without the need for recombination with an endogenous plasmid, indicating the potential probability of horizontal chlamydial pathogenic gene transfer by plasmids between chlamydial species.

## INTRODUCTION

Obligate intracellular chlamydiae infect humans and animals, causing a wide range of different diseases (1). Two species of *Chlamydia* are major human pathogens: Chlamydia trachomatis is the most common bacterial sexually transmitted disease (STD) worldwide and the leading cause of infectious blindness (1), and C. pneumoniae causes acute and chronic infections of the upper and lower respiratory tracts and is associated with cardiovascular diseases (2). The phylogenetic tree of C. pneumoniae genome sequences shows a clear separation of vascular C. pneumoniae isolates from respiratory isolates, indicating a specific tissue tropism in humans (3).

Interestingly and in contrast to C. trachomatis, C. pneumoniae is widely distributed in different animals, such as horses, koalas, and the western barred bandicoot, as well as amphibians and reptiles (1, 3–5), causing ocular, urogenital, and respiratory tract infections (5, 6).

All *Chlamydia* spp. share a developmental cycle that alternates between two distinct bacterial forms, infectious elementary bodies (EBs) and the replicating reticulate bodies (RBs) (2).

Although plasmids have never been found in any human isolates of C. pneumoniae, most *Chlamydia* spp., including animal isolates of C. pneumoniae, carry plasmids sized approximately 7.5 kb. The plasmid contains eight putative coding DNA sequences (CDS1 to CDS8) (7). CDS1 and CDS2 possibly regulate plasmid replication (7), whereas CDS3, CDS4, and CDS8 play a pivotal role in plasmid maintenance (7). CDS2 also contains an antisense small RNA (sRNA-2), which is necessary for plasmid maintenance (7). Moreover, CDS5 acts as a virulence factor in C. trachomatis- or C. muridarum-infected mice (7). It has also been shown that some chromosomal genes, for example, the glycogen synthase gene *glgA*, are regulated by proteins encoded by CDS6 or CDS7 (7).

Tools for genetic manipulation, such as a plasmid shuttle vector system of C. trachomatis, have only recently become available (8–10). The utilization of C. trachomatis-derived plasmid shuttle vectors has allowed the introduction of plasmids into C. trachomatis (8, 11).

Although plasmid shuttle vector pGFP::SW2 derived from C. trachomatis serovar E can be transformed into other C. trachomatis serovars (8–10, 12, 13), transformation into other species such as C. muridarum has not been successful, indicating the presence of a species barrier for transformation and/or replication (14). Importantly, Wang et al. demonstrated that the barrier was encountered not at the plasmid acquisition stage but rather during plasmid replication or maintenance (15). In addition, they showed that CDS2 was required for plasmid maintenance, thus making it a significant determinant of plasmid tropism (15).

In our study, we describe for the first time a robust, reproducible transformation protocol for human as well as animal isolates of C. pneumoniae. It uses an animal C. pneumoniae-derived plasmid shuttle vector, and transformation results in stable transformants that can grow in the absence of selection. In addition, we demonstrate that the species barrier in chlamydial transformation between C. pneumoniae and C. felis (16–18) can be overcome.

## RESULTS

### Selection criteria and vector design for transformation of C. pneumoniae.

The 7,368-bp plasmid pCpnE1 (7,368 bp; GenBank accession no. X82078.1) was isolated from plasmid-bearing C. pneumoniae N16 and cloned into vector pRSGFPCAT. We verified the transformation method in C. trachomatis using pGFP::SW2 (8). We therefore used a similar design strategy for the construction of a C. pneumoniae-derived plasmid shuttle vector.

**FIG 1:**
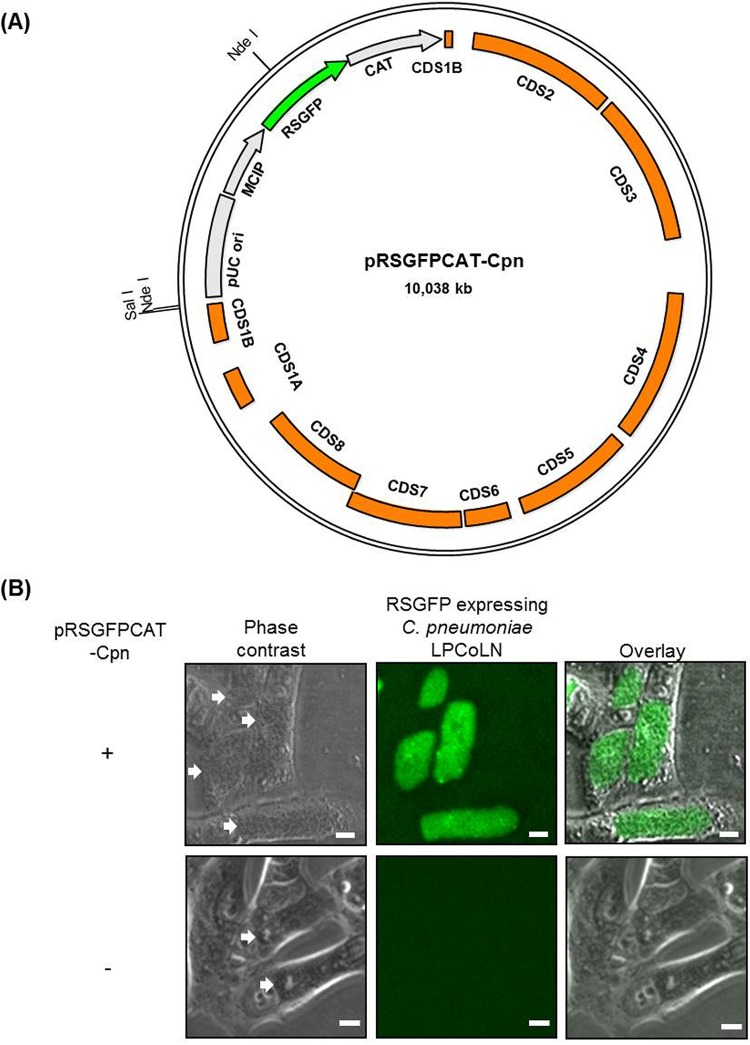
Map of the C. pneumoniae*-*derived shuttle vector pRSGFPCAT-Cpn and the RSGFP expression in C. pneumoniae LPCoLN. (A) The CDSs of pCpnE1 and pRSGFPCAT are shown in orange and light gray, respectively. The red-shifted green fluorescent protein gene (RSGFP) is shown in green. MCIP, meningococcal class I protein promoter; CAT, chloramphenicol acetyltransferase gene. (B) pRSGFPCAT-Cpn-transformed and untransformed C. pneumoniae LPCoLN. Transformed (+) C. pneumoniae was grown in HEp-2 cells with chloramphenicol, and untransformed (−) C. pneumoniae was grown without chloramphenicol for 48 h. GFP fluorescence of chlamydial inclusions was visualized in living cells without fixing and staining. Images are representative of three independent experiments. White arrows show chlamydial inclusions. Bars, 10 µm.

NdeI-cleaved pCpnE1 was ligated into VspI-digested pRSGFPCAT, resulting in pRSGFPCAT-Cpn (Fig. 1A). We confirmed the expected fragment sizes by digestion of pRSGFPCAT-Cpn with NdeI (1,476-bp and 8,562-bp fragments) and SalI (10,038-bp fragment) (see Fig. S1A in the supplemental material) in addition to sequencing of pRSGFPCAT-Cpn (Fig. S1B).

10.1128/mSphere.00412-18.1 FIG S1 The digestion and the nucleotide sequence of pRSGFPCAT-Cpn. (A) Digestion of pRSGFPCAT-Cpn with NdeI and SalI. (B) The nucleotide sequence of pRSGFPCAT-Cpn. pCpnE1 is shown by gray. pRSGFPCAT is shown in white. Three additional (green) or three different (red) nucleotides compared to original sequence of pCpnE1 (GenBank accession no. X82078.1). Six nucleotides, AATTCG, are missing in this sequence compared to the sequence of GenBank entry X82078.1. The region of pRSGFPCAT is 100% identical compared to the previous report. The region of pCpnE1 is 99.998% identical compared to original sequence of pCpnE1 (GenBank accession no. X82078.1). The sequence presented here is correct for vector pRSGFPCAT-Cpn shown in this study. Download FIG S1, TIF file, 0.7 MB. Copyright © 2018 Shima et al. 2018 Shima et al. This content is distributed under the terms of the Creative Commons Attribution 4.0 International license.

Since no viable culture of the parent strain of C. pneumoniae N16 was available to us, we investigated whether we could utilize a different animal-isolate plasmid-bearing C. pneumoniae strain for the transformation. In the plasmid sequence comparison, more than 94% of total CDSs were identical between pCpnE1 derived from C. pneumoniae N16 and pCpnKo derived from C. pneumoniae LPCoLN, indicating highly conserved plasmid sequences (Table S1). We considered that the high homology of plasmid sequences between these two C. pneumoniae plasmids gave the optimal chance of success, and thus, we selected C. pneumoniae LPCoLN as the recipient C. pneumoniae strain to perform our initial transformation.

10.1128/mSphere.00412-18.5 TABLE S1 Sequence homology between C. pneumoniae N16 and other *Chlamydia* spp. Download Table S1, DOCX file, 0.02 MB. Copyright © 2018 Shima et al. 2018 Shima et al. This content is distributed under the terms of the Creative Commons Attribution 4.0 International license.

The wild-type untransformed C. pneumoniae LPCoLN inclusions have no naturally occurring fluorescence; in contrast, a strong red-shifted green fluorescent protein (RSGFP) signal was detected in C. pneumoniae LPCoLN (C. pneumoniae LPCoLN-pRSGFPCAT-Cpn) inclusions after transformation of this recipient with the recombinant shuttle vector and infection in HEp-2 cells (Fig. 1B).

### The properties of pRSGFPCAT-Cpn-transformed C. pneumoniae LPCoLN.

**FIG 2:**
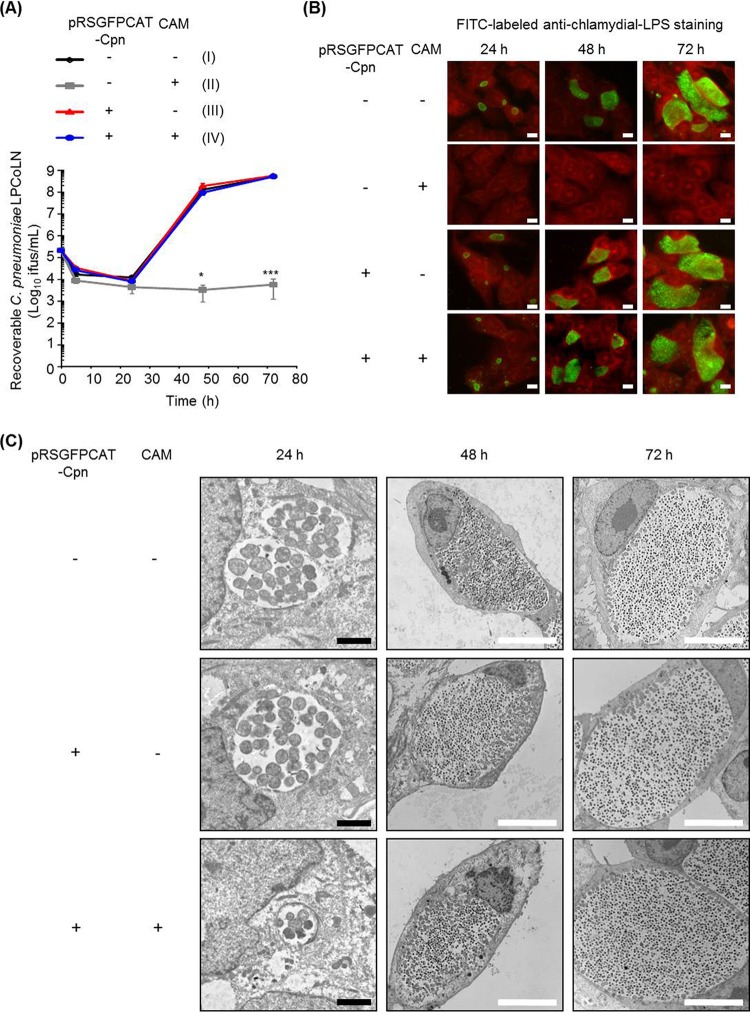
One-step growth curve and the inclusion morphology of pRSGFPCAT-Cpn-transformed and untransformed C. pneumoniae LPCoLN. (A) Recoverable C. pneumoniae at 5, 24, 48, and 72 hpi. pRSGFPCAT-Cpn-transformed and untransformed C. pneumoniae bacteria were grown in HEp-2 cells with or without chloramphenicol. The numbers of recoverable C. pneumoniae bacteria under each condition (II, III, and IV) at the indicated time were compared to those of untransformed C. pneumoniae without chloramphenicol (I) (*n* = 3, mean ± SEM; Sidak’s multiple comparison; *, *P* ≤ 0.05; ***, *P* ≤ 0.001). (B) Representative immunofluorescence images of pRSGFPCAT-Cpn-transformed and untransformed C. pneumoniae at 24, 48, and 72 hpi. Chlamydial inclusions were stained by FITC-labeled monoclonal chlamydial-LPS antibodies. Evans blue counterstaining of host cells was used for better characterization of intracellular inclusions. Images are representative of three independent experiments. Bars, 10 µm. (C) pRSGFPCAT-Cpn-transformed and untransformed C. pneumoniae bacteria with or without chloramphenicol treatment were analyzed with a TEM at 24, 48, and 72 hpi. Black bars, 2 µm; white bars, 10 µm.

We investigated whether the transformation of C. pneumoniae LPCoLN using pRSGFPCAT-Cpn influenced chlamydial growth and morphological characteristics (Fig. 2). C. pneumoniae LPCoLN-pRSGFPCAT-Cpn showed similar growth characteristics in the absence or presence of chloramphenicol (CAM) as wild-type C. pneumoniae LPCoLN (Fig. 2A). In addition to growth characteristics, both immunofluorescence and transmission electron microscopic (TEM) analyses revealed similar inclusion morphologies between the wild type and C. pneumoniae LPCoLN-pRSGFPCAT-Cpn (Fig. 2B and C).

**FIG 3:**
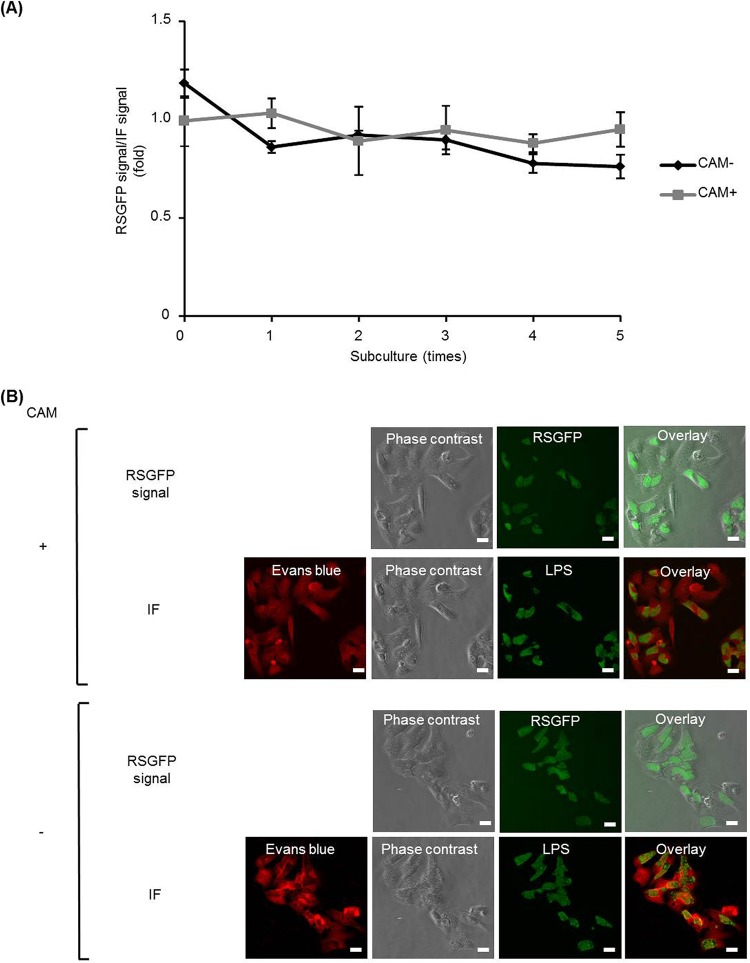
The pRSGFPCAT-Cpn plasmid can be stably retained in C. pneumoniae LPCoLN and expresses RSGFP. (A) pRSGFPCAT-Cpn-transformed C. pneumoniae was subcultured in HEp-2 cells with and without chloramphenicol every 3 to 4 days over 5 passages. The ratio of live RSGFP expressed in inclusions to immunofluorescence staining (IF) of chlamydial inclusions by mouse anti-chlamydial LPS antibody was calculated. No statistically significant difference by one-way analysis of variance was found (*n* = 3, mean ± SEM, Sidak’s multiple comparison). (B) Representative RSGFP and immunofluorescence images of pRSGFPCAT-Cpn-transformed C. pneumoniae LPCoLN 48 hpi at passage 5. After an RSGFP signal was detected by fluorescence microscopy, the cells were fixed by methanol and chlamydial inclusions were stained by FITC-labeled monoclonal chlamydial-LPS antibodies. Evans blue counterstaining of host cells was used for better characterization of intracellular inclusions. Bars, 20 µm.

The plasmid stability can be assessed by the ratio of RSGFP-expressing inclusions to antichlamydial lipopolysaccharide (LPS) antibody-stained inclusions. As shown, the ratio of live RSGFP-expressing inclusions to immunofluorescence staining of chlamydial inclusions showed no significant difference (Fig. 3A and B). This indicates that the pRSGFPCAT-Cpn plasmid could be stably retained over five passages of C. pneumoniae LPCoLN-pRSGFPCAT-Cpn even in the absence of CAM. Taken together, these data indicate that C. pneumoniae LPCoLN-pRSGFPCAT-Cpn is stable and the presence of the shuttle vector does not affect the growth and morphology of C. pneumoniae LPCoLN.

### RSGFP is expressed in human isolates of C. pneumoniae.

We next investigated whether transformation was possible in a plasmid-free human isolate of C. pneumoniae. Since we found unique genotypes in cardiovascular isolates (CVs) (3) and characterization of CVs has been the focus of our recent research, C. pneumoniae CV-6 isolated from coronary arteries (19, 20) was selected in this study. We first compared the whole-genome sequence of C. pneumoniae CV-6 to the previously reported sequence of cardiovascular isolate C. pneumoniae CV-14. The genome sequence revealed that C. pneumoniae CV-6 is >99.99829% identical to previously sequenced cardiovascular C. pneumoniae CV-14. Only 8 single nucleotide polymorphisms (SNPs), 2 insertions and deletions (indels), and no inversions were found in C. pneumoniae CV-6 compared to C. pneumoniae CV-14, indicating high homology of genome sequences within different cardiovascular isolates (Table S2) (3).

10.1128/mSphere.00412-18.6 TABLE S2 Whole-genome sequence comparison between C. pneumoniae CV-14 and C. pneumoniae CV-6. Download Table S2, DOCX file, 0.01 MB. Copyright © 2018 Shima et al. 2018 Shima et al. This content is distributed under the terms of the Creative Commons Attribution 4.0 International license.

**FIG 4:**
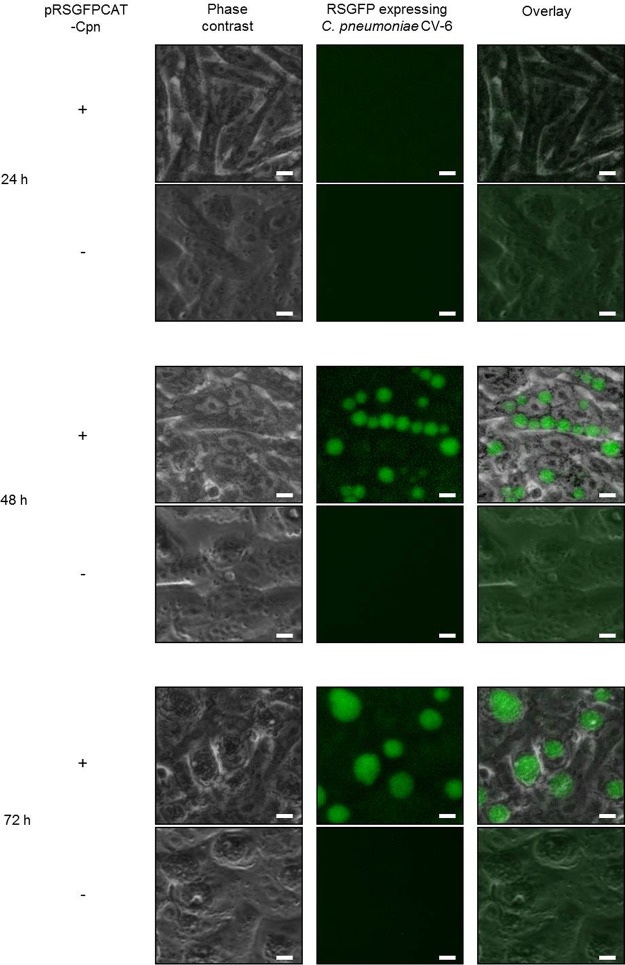
RSGFP expression in pRSGFPCAT-Cpn-transformed human cardiovascular isolate C. pneumoniae CV-6. pRSGFPCAT-Cpn-transformed (+) C. pneumoniae CV-6 was grown in HEp-2 cells with chloramphenicol, and untransformed (−) C. pneumoniae was grown without chloramphenicol for 24, 48, and 72 h. GFP fluorescence of chlamydial inclusions was visualized in living cells without fixing and staining. Images are representative of three independent experiments. Bars, 10 µm.

At 48 and 72 h postinfection (hpi), a strong RSGFP signal was detected in inclusions of the successful transformant of C. pneumoniae CV-6 (C. pneumoniae CV-6-pRSGFPCAT-Cpn) (Fig. 4 and Table 1). When we further performed genome sequencing analysis of C. pneumoniae CV-6-pRSGFPCAT-Cpn, >99.99902% of the whole genome was identical between wild-type C. pneumoniae CV-6 and C. pneumoniae CV-6-pRSGFPCAT-Cpn. Moreover, only 9 SNPs, no indels, and no inversions were found in C. pneumoniae CV-6-pRSGFPCAT-Cpn compared to wild-type C. pneumoniae CV-6, excluding the possibility of contamination with other chlamydial isolates (Table S3). In addition, the sequence of pRSGFPCAT-Cpn of C. pneumoniae CV-6-pRSGFPCAT-Cpn was 100% identical, indicating no insertions and deletions (indels) and no inversions compared to the original shuttle vector pRSGFPCAT-Cpn.

**TABLE 1:** RSGFP expression in different chlamydial species

Species	Strain	Host	Presence of wild-type plasmid^*a*^	Expression of RSGFP (pRSGFPCAT-Cpn)^*b*^
C. pneumoniae	CV-6	Human	−	+
	IOL-207	Human	−	+
	LPCoLN	Koala	+	+
C. felis	Cello	Cat	+	+
	Not identified (N.I.)	N.I.	+	+
	02DC26 (Cf02-23)	Cat	−	+
C. trachomatis	L2 (25667R)	Human	−	−
C. muridarum	MoPn/Nigg	Mouse	+	−
C. abortus	C18/98 (B577)	Sheep	−	−
C. caviae	03DC25 (GPIC)	Guinea pig	+	−
C. pecorum	14DC102 (E58)	Cow	−	−

a Plasmid-bearing strain (+) and plasmid-free strain (−).

b RSGFP signal positive (+) and negative (−).

10.1128/mSphere.00412-18.7 TABLE S3 Whole-genome sequence comparison between wild-type C. pneumoniae CV-6 and C. pneumoniae CV-6-pRSGFPCAT-Cpn. Download Table S3, DOCX file, 0.01 MB. Copyright © 2018 Shima et al. 2018 Shima et al. This content is distributed under the terms of the Creative Commons Attribution 4.0 International license.

**FIG 5:**
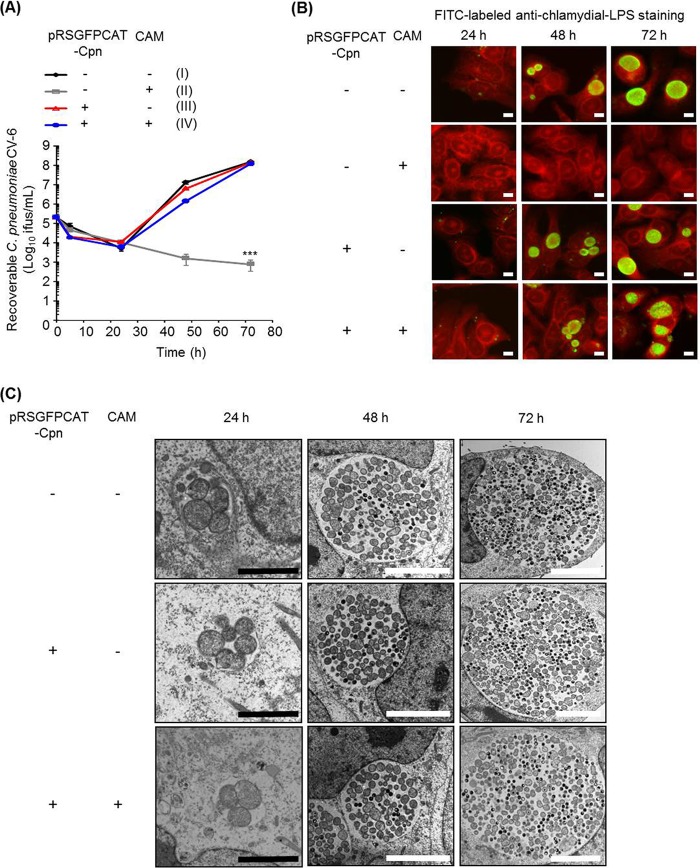
One-step growth curve and the inclusion morphology of pRSGFPCAT-Cpn-transformed and untransformed C. pneumoniae CV-6. (A) Recoverable C. pneumoniae at 5, 24, 48, and 72 hpi. pRSGFPCAT-Cpn-transformed and untransformed C. pneumoniae bacteria were grown in HEp-2 cells with or without chloramphenicol. The numbers of recoverable C. pneumoniae bacteria under each condition (II, III, and IV) at the indicated time were compared to those of untransformed C. pneumoniae without chloramphenicol (I) (*n* = 4, mean ± SEM; Sidak’s multiple comparison, ***, *P* ≤ 0.001). (B) Representative immunofluorescence images of pRSGFPCAT-Cpn-transformed and untransformed C. pneumoniae at 24, 48, and 72 hpi. Chlamydial inclusions were stained by FITC-labeled monoclonal chlamydial-LPS antibodies. Evans blue counterstaining of host cells was used for better characterization of intracellular inclusions. Images are representative of four independent experiments. Bars, 10 µm. (C) pRSGFPCAT-Cpn-transformed and untransformed C. pneumoniae bacteria with or without chloramphenicol treatment were analyzed with a TEM at 24, 48, and 72 hpi. Black bars, 2 µm; white bars, 5 µm.

**FIG 6:**
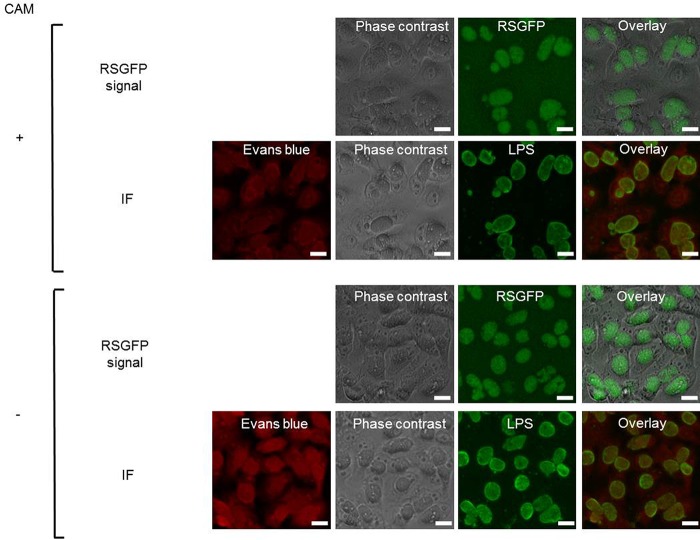
The pRSGFPCAT-Cpn plasmid can be stably retained in C. pneumoniae CV-6 and expresses RSGFP. pRSGFPCAT-Cpn-transformed C. pneumoniae was subcultured in HEp-2 cells with and without chloramphenicol every 2 to 3 days. Representative RSGFP and immunofluorescence staining (IF) images of pRSGFPCAT-Cpn-transformed C. pneumoniae CV-6 were taken 72 hpi at passage 5. After an RSGFP signal was detected by fluorescence microscopy, the cells were fixed by methanol and chlamydial inclusions were stained by FITC-labeled monoclonal chlamydial-LPS antibodies. Evans blue counterstaining of host cells was used for better characterization of intracellular inclusions. Bars, 20 µm.

Furthermore, C. pneumoniae CV-6-pRSGFPCAT-Cpn showed similar growth characteristics and chlamydial morphology in the absence or presence of CAM as wild-type C. pneumoniae CV-6 (Fig. 5A to C). Stable plasmid retention with and without CAM treatment was also observed in C. pneumoniae CV-6-pRSGFPCAT-Cpn (Fig. 6).

These results were repeated in three independent experiments and demonstrate the successful transformation of pRSGFPCAT-Cpn into a human cardiovascular isolate of C. pneumoniae.

The reproducibility of the protocol was confirmed by the successful transformation of the human C. pneumoniae IOL-207 isolate that is associated with community-acquired pneumonia using the same method with pRSGFPCAT-Cpn in the Southampton lab (Fig. S2).

10.1128/mSphere.00412-18.2 FIG S2 The RSGFP expression in pRSGFPCAT-Cpn-transformed human isolate C. pneumoniae IOL-207. pRSGFPCAT-Cpn-transformed C. pneumoniae IOL-207 was grown in McCoy cells with chloramphenicol for 72 h. GFP fluorescence of chlamydial inclusions was visualized in living cells without fixing and staining. Bars, 10 µm. Download FIG S2, TIF file, 1.3 MB. Copyright © 2018 Shima et al. 2018 Shima et al. This content is distributed under the terms of the Creative Commons Attribution 4.0 International license.

### The C. pneumoniae-derived plasmid shuttle vector pRSGFPCAT-Cpn is able to cross the species barrier.

Species barriers for the plasmid-mediated transformation between different *Chlamydia* species have been reported (14, 15). Understanding the nature of this phenomenon is important for improving the repertoire of tools needed for the genetic modification of chlamydiae, particularly for horizontal gene transfer between different chlamydial species. Therefore, we investigated the limits of potential species barriers with our shuttle vector.

Since a functional species-matched CDS2 is essential (supplied by the recipient host) in forcing a C. trachomatis plasmid to replicate in C. muridarum (15), we selected different plasmid-bearing *Chlamydia* spp. such as C. muridarum, C. caviae, and C. felis for the transformation experiments. Furthermore, we also expanded the investigation to plasmid-free *Chlamydia* spp. such as C. trachomatis L2 (25667R), C. pecorum, and C. abortus, but we had to exclude C. psittaci due to biosafety considerations.

**FIG 7:**
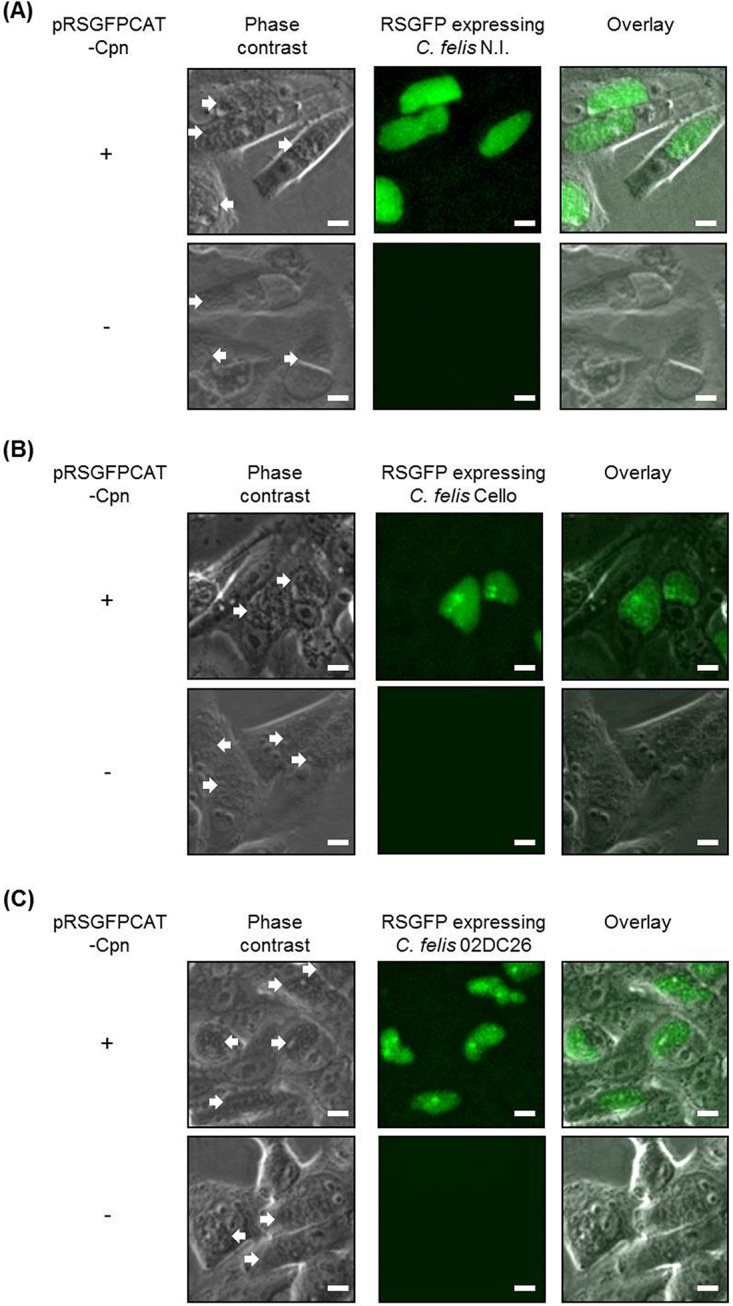
The RSGFP expression in different C. felis strains. pRSGFPCAT-Cpn-transformed and untransformed C. felis strains, C. felis N.I. (A), C. felis Cello (B), and C. felis 02DC26 (Cf02-23) (C). Transformed (+) C. felis strains were grown in HEp-2 cells with chloramphenicol, and untransformed (−) C. felis strains were grown without chloramphenicol for 48 h. GFP fluorescence of chlamydial inclusions was visualized in living cells without fixing and staining. Images are representative of three independent experiments. Arrows show chlamydial inclusions. Bars, 10 µm.

In accordance with previous observations, we did not obtain RSGFP-fluorescent inclusions in plasmid-bearing *Chlamydia* spp. such as C. muridarum and C. caviae, as well as plasmid-free *Chlamydia* spp. including C. trachomatis, C. pecorum, and C. abortus. In contrast, a strong RSGFP signal was observed in plasmid-bearing C. felis strain N.I. (for “not identified”) (Fig. 7A and Fig. S3), which is 99.98533% identical in the whole genome and 100% identical in plasmid sequence to C. felis Fe/C-56 (Table S4). To investigate whether this was a serendipitous finding for a single strain or whether it was a new novel tropism for the transformative C. pneumoniae plasmid, we tested another plasmid-bearing C. felis isolate, C. felis Cello (Fig. 7B and Fig. S3). In addition to the successful transformation of plasmid-bearing C. felis N.I. and Cello, the naturally occurring plasmid-free C. felis strain 02DC26 could also be transformed using pRSGFPCAT-Cpn (Fig. 7C and Fig. S3). These results indicate that there is no barrier to selecting recombinants within the distinct and separate species C. felis. To investigate whether this was a plasmid-related or host-related phenomenon, we checked if our C. felis strains could be transformed by C. trachomatis plasmid backbone pGFP::SW2. None of these C. felis isolates (C. felis N.I., C. felis Cello, and C. felis strain 02DC26) could be transformed by the C. trachomatis shuttle vector pGFP::SW2 (unpublished observation).

10.1128/mSphere.00412-18.3 FIG S3 PCR analysis revealing the plasmid-bearing C. felis strains. Two different primer sets, pCFelis-F and pCFelis-R for lanes 2 to 6 and pCF01-F and pCF01-R for lanes 7 to 11, were used to confirm whether each strain is plasmid positive or negative. Lanes: 1, 100-bp DNA ladder; 2 and 7, C. felis N.I.; 3 and 8, C. felis 02DC26; 4 and 9, C. felis Cello; 5 and 10, pRSGFPCAT-Cpn shuttle vector; 6 and 11, negative control (water). Download FIG S3, TIF file, 0.1 MB. Copyright © 2018 Shima et al. 2018 Shima et al. This content is distributed under the terms of the Creative Commons Attribution 4.0 International license.

10.1128/mSphere.00412-18.8 TABLE S4 Whole-genome sequence comparison between C. felis Fe/C-56 and C. felis N.I. Download Table S4, DOCX file, 0.01 MB. Copyright © 2018 Shima et al. 2018 Shima et al. This content is distributed under the terms of the Creative Commons Attribution 4.0 International license.

Wang et al. demonstrated that CDS2 with short adjoining sequences in the chlamydial plasmid is a major determinant of plasmid tropism (15). Therefore, we investigated whether a recombination between the wild-type C. felis plasmid and the pCpnE1 region of pRSGFPCAT-Cpn took place in the pRSGFPCAT-Cpn-transformed C. felis Cello. The sequence analysis revealed 100% identity between pRSGFPCAT-Cpn isolated from C. felis Cello and one from C. pneumoniae, indicating no recombination between pRSGFPCAT-Cpn and a wild-type plasmid from C. felis Cello (Fig. S1B and S4). These data indicate that plasmid species barriers can be exceptionally crossed in C. felis species without a recombination between plasmids of different species.

10.1128/mSphere.00412-18.4 FIG S4 Map of pRSGFPCAT-Cpn that was isolated from RSGFP expressed in C. felis Cello. The CDSs of pCpnE1 are shown in red. The red-shifted green fluorescent protein gene (RSGFP) is shown in green. MCIP, meningococcal class I protein promoter; CAT, chloramphenicol acetyltransferase gene. The pRSGFPCAT region is 100% identical and the pCpnE1 region is 99.6% identical to original sequences. CDSs 1 to 4, 7, and 8 are 100% identical to those of pCpnE1. Only 2 bp in CDS5 and 1 bp in CDS6 are mismatches of those of pCpnE1 (GenBank accession no. X82078.1). Download FIG S4, TIF file, 0.9 MB. Copyright © 2018 Shima et al. 2018 Shima et al. This content is distributed under the terms of the Creative Commons Attribution 4.0 International license.

## DISCUSSION

The C. trachomatis-derived plasmid was modified and developed as a plasmid shuttle vector for use as a transformation tool in recent years (8). This plasmid shuttle vector system has been broadly used in studies of C. trachomatis and C. muridarum (21). However, it was not known whether this system could be utilized in C. pneumoniae or in other chlamydial species.

In this study, we demonstrated that the C. pneumoniae plasmid shuttle vector pRSGFPCAT-Cpn was stably retained not only in an animal isolate of C. pneumoniae but also in different human isolates of C. pneumoniae. In a previous study, we found that cardiovascular isolates of C. pneumoniae were genetically closely related and could be distinguished from the respiratory tract isolates on the basis of unique nonsynonymous SNPs (nsSNPs) (3). Importantly, these nsSNPs are located in genes involved in chlamydial RB-to-EB transition, inclusion membrane formation, bacterial stress response, and metabolism (3). Therefore, the ultimate aim of transformation in cardiovascular isolates of C. pneumoniae is to decipher the functions of such genes that might be linked to tissue tropism.

Human and animal C. pneumoniae isolates can be distinguished by the presence or absence of plasmids, respectively (22–25). While plasmids have never been found in any human isolates of C. pneumoniae studied, animal isolates of C. pneumoniae harbor plasmids that are similar to the plasmids from other *Chlamydia* spp. in nucleotide sequence, size, and gene organization (4, 22–24). In addition, whole-genome analysis revealed 6,213 SNPs (3,298 synonymous and 2,915 nonsynonymous) between animal isolate C. pneumoniae and human isolate C. pneumoniae (4). Importantly, human isolates of C. pneumoniae have evolved from animal isolates that have adapted to humans through fragmentation and decay of functional genes (22, 26). Since plasmids are also not essential for infection and survival, they might have been lost in human isolates during evolutional processes (22). Considering evolutionary linkage between animal isolates and human isolates of C. pneumoniae, we suggest that the equine C. pneumoniae-derived plasmid shuttle vector pRSGFPCAT-Cpn can replicate in plasmid-free human isolates of C. pneumoniae.

A range of antimicrobials have been used for the selection of transformed *Chlamydia* spp. (8, 10, 13). β-Lactam antimicrobial treatment is commonly and successfully used for selection of tranformants of C. trachomatis. However, while this is permissible for urogenital isolates, there are no guidelines from the U.S. National Institutes of Health (NIH) for respiratory chlamydiae (21). Therefore, chloramphenicol was chosen to select pRSGFPCAT-Cpn-transformed *Chlamydia* spp.

There is one report that a C. trachomatis-derived plasmid shuttle vector was introduced into different chlamydial species (27). However, Song et al. demonstrated that *Chlamydia* spp. could be transformed when the plasmid shuttle vector was constructed with the same backbone as the plasmid harbored in the identical species (14). Furthermore, Wang et al. showed that recombination of the CDS2 region was required for stable replication in a new host species and that this sequence is a determinant of plasmid tropism (15). Along with other research groups (14, 15), we were unable to transform the C. trachomatis plasmid to other chlamydial species including C. pneumoniae under various conditions. Therefore, it was crucial for us to construct a C. pneumoniae-derived plasmid shuttle vector for transformation of C. pneumoniae.

Similar plasmid tropism was also observed using the plasmid shuttle vector pRSGFPCAT-Cpn. We did not observe an RSGFP signal in the *Chlamydia* species C. trachomatis, C. muridarum, C. caviae, C. abortus, and C. pecorum when transformations were attempted using the C. pneumoniae pCpnE1 backbone plasmid shuttle vector pRSGFPCAT-Cpn. In contrast, three different C. felis strains were stably transformed by pRSGFPCAT-Cpn. The sequencing showed no changes in the transforming shuttle vector, confirming that it had not recombined with the endogenous plasmid. Thus, we show that plasmid recombination is not necessary to cross the species barrier in C. felis.

Although most C. felis strains, including strains Cello and Pring, harbor plasmids, a few, including the Baker strain, lack a plasmid (16, 18). Since we could observe strong RSGFP signals from plasmid-harboring or plasmid-free C. felis strains, we conclude that the presence or absence of a wild-type plasmid does not influence transformation of C. felis. C. felis strains could be transformed only by C. pneumoniae backbone plasmid shuttle vector pRSGFPCAT-Cpn but not by the C. trachomatis backbone plasmid shuttle vector pGFP::SW2, although both posssess approximately 60% homologies compared with the plasmid sequence of C. felis N.I. (see Table S5 in the supplemental material). This indicates that an as-yet-unknown biological property of the C. pneumoniae plasmid is shared with the C. felis plasmid.

10.1128/mSphere.00412-18.9 TABLE S5 Sequence homology among C. felis N.I., C. trachomatis, and C. pneumoniae. Download Table S5, DOCX file, 0.01 MB. Copyright © 2018 Shima et al. 2018 Shima et al. This content is distributed under the terms of the Creative Commons Attribution 4.0 International license.

Horizontal gene transfer plays a crucial role in bacterial genome evolution and adaptation to environmental stresses (28). In forced coinfections of cells in culture, Suchland et al. showed fusion of inclusions between different chlamydial species such as C. suis and C. muridarum, indicating the possibility of direct contact between chlamydial species in host cells (29). Furthermore, gene recombination, such as insertion of antibiotic-resistant genes into the chlamydial genome, has been observed not only from strain to strain within species but also between *Chlamydia* species *in vitro* (29, 30). Our data indicate that horizontal gene transfer via plasmids is possible between some chlamydial species, in contrast to previous suggestions (31). C. felis is a chlamydial species that causes pneumonia and conjunctivitis in cats (16–18). If one considers the high proportion of households owning cats worldwide (approximately one-third of all households) (32, 33), horizontal gene transfer between C. pneumoniae and C. felis may even be relevant for public health.

Taken together, our findings indicate that the C. pneumoniae pCpnE1 backbone plasmid shuttle vector pRSGFPCAT-Cpn is a useful C. pneumoniae transformation tool. Critically, the methods are reproducible, having been repeated in two independent laboratories. Since various chlamydial mutagenesis methods have been developed recently (21), their combination with the C. pneumoniae shuttle vector system will enable more precise elucidation of pathogenic factors and mechanisms underlying C. pneumoniae infection.

## MATERIALS AND METHODS

### Bacterial strains, epithelial cells, and chemicals.

Chlamydial strains which were used for transformation are listed in Table 1. The cardiovascular C. pneumoniae strain CV-6 was provided by Matthias Maass in 1998 (University of Luebeck, Luebeck, Germany) (19, 20). C. pneumoniae IOL-207 was donated by John Treharne in 1990 (Institute of Ophthalmology, London, United Kingdom) (34–38). C. pneumoniae strains CV-6 and IOL-207 came from anonymized patients, for whom records no longer exist as the strains were isolated before 1998 and 1972, respectively (38). Concerning the ethics regulations at the University of Luebeck, this approach was feasible at that time without informed consent and was recently updated by an opt-out regulation that allows basic research with clinical isolates including minimal parametric data from the patient as long as not otherwise indicated by the patient on admission. The usage of clinical isolates that have been collected before the opt-out regulation has been approved by the local ethics committee when the reference of the initial publication is given and no further clinical data from the patient are given. Furthermore, with respect to the isolates CV-6 and IOL-207, these are anonymized, and therefore no data entry exists that allows access to the original patient information. C. trachomatis strain L2 (25667R) was kindly provided from the already existing bacterial collection of Luis de la Maza (University of California, Irvine, CA, USA) (39).

The following animal strains were provided by the National and OIE Reference Laboratory for Chlamydioses at FLI Jena: C. felis 02DC26 (Cf02-23), C. muridarum MoPn/Nigg (DSM-28544), C. abortus C18/98 (B577, DSM-27654), C. caviae 03DC25 (GPIC, DSM-19441), and C. pecorum 14DC102 (E58, DSM-29919). C. pneumoniae LPCoLN came from the bacterial collection of Peter Timms (University of Sunshine Coast, Maroochydore, Australia) (4). C. felis strains came from the bacterial collection of Ian N. Clarke (University of Southampton, Southampton, United Kingdom). To confirm the species identity and rule out mixed cultures, genomic DNA of all strains was examined using a DNA microarray as described previously (40). Escherichia coli DH5α and Dam and Dcm methylase-deficient strain JM110 (Agilent Technologies, Santa Clara, CA, USA) were used to construct and manipulate plasmids. HEp-2 cells (ATCC CCL-23) or McCoy cells (European Collection of Authenticated Cell Cultures 90010305) were used for the growth of chlamydiae. All chemicals were purchased from Sigma-Aldrich (Deisenhofen, Germany).

### Construction of C. pneumoniae-derived plasmid shuttle vector.

The plasmid vector pRSGFPCAT was constructed as described previously (8). In brief, 2,670-bp pRSGFPCAT contains the pUC origin and the RSGFP-chloramphenicol acetyltransferase (CAT) gene fusion which were regulated by the meningococcal class I protein promoter (MCIP) derived from Neisseria meningitidis MC50 (8). C. pneumoniae N16 plasmid pCpnE1 (7,368 bp; GenBank accession no. X82078.1) was cleaved by NdeI and ligated into VspI-digested pRSGFPCAT, resulting in pRSGFPCAT-Cpn.

### Cell culture.

HEp-2 cells in 1 ml or 4 ml Dulbecco's modified Eagle medium (DMEM) supplemented with 10% fetal bovine serum (FBS) (Invitrogen), 1 mM sodium pyruvate (Pan-Biotech GmbH, Aidenbach, Germany), and 30 mM HEPES were seeded into 24- or 6-well plates (Greiner Bio-One, Frickenhausen, Germany) and cultured overnight at 37°C under 5% CO_2_. Cells treated with 1 µg/ml cycloheximide were infected with chlamydiae by centrifugation at 700 × *g* for 1 h at 35°C.

### Genetic transformation.

Transformation was performed as described in the previous study with minor modifications (8). pRSGFPCAT-Cpn was isolated from Dam and Dcm methylase-deficient strain JM110 with the Qiagen plasmid megakit (Hilden, Germany). Following optimization, subinhibitory concentrations of CAM (0.2 to 0.5 µg/ml) were used for the selection of transformed chlamydiae.

A 1 × 10^7^-inclusion-forming-unit (IFU) quantity of chlamydiae and 6 to 15 µg of pRSGFPCAT-Cpn were incubated in 200 µl calcium chloride buffer (10 mM Tris, 50 mM calcium chloride, pH 7.4) for 30 min at room temperature. Then, 200 µl of chlamydiae mixed with pRSGFPCAT-Cpn was incubated with 200 µl of trypsinized HEp-2 cells (4 × 10^6^) in calcium chloride buffer for 20 min with mild agitation.

The total volume, 100 µl, of the mixture of HEp-2 cells, pRSGFPCAT-Cpn, and chlamydiae was added to each well containing 2 ml DMEM supplemented with 10% FBS, 1 mM sodium pyruvate, 30 mM HEPES, and 1 µg/ml cycloheximide in a 6-well plate and incubated for 72 h at 37°C under 5% CO_2_. To increase the infection rate for the initial culture, the monolayer of 1.4 × 10^6^ HEp-2 cells per well in a 6-well plate was also used, and infected cells were cultured at 37°C under a 5% CO_2_ and 20 or 2% O_2_ atmosphere. Infected HEp-2 cells were scraped and lysed with glass beads.

Freshly prepared HEp-2 cells were infected with transformed or untransformed chlamydiae in 6-well plates containing 5 ml DMEM supplemented with 10% FBS, 1 mM sodium pyruvate, 30 mM HEPES, 1 µg/ml cycloheximide, and 0.2 to 0.5 µg/ml CAM. Medium was changed every 72 h, and passages were performed every 3 to 7 days. If an RSGFP signal was not observed in chlamydial inclusions within 40 days or by passage 5, we designated those strains “not transformed.”

### Recovery assay.

Cells were infected with either untransformed or pRSGFPCAT-Cpn-transformed C. pneumoniae strains at 0.5 IFU/cell. Cycloheximide (1 µg/ml) and CAM with concentrations of 0.8 µg/ml (C. pneumoniae LPCoLN) and 1.1 µg/ml (C. pneumoniae CV-6) were used in chlamydial infection. The plate was further centrifuged at 700 × *g* for 1 h at 35°C and incubated for 5, 24, 48, and 72 h. After the indicated time points, the cells were subsequently cultured for 48 h for determination of the recoverable C. pneumoniae.

### Plasmid stability.

HEp-2 cells were initially infected with pRSGFPCAT-Cpn-transformed C. pneumoniae LPCoLN or CV-6 at 0.5 IFU/cell in the presence or absence of CAM (0.8 µg/ml for C. pneumoniae LPCoLN and 1.1 µg/ml for C. pneumoniae CV-6) under standard conditions. The infected cells were subcultured every 2 to 4 days for 5 times. From the second subculture, HEp-2 cells were infected with serial dilutions of C. pneumoniae at 0.5 to 0.8 IFU/cell. After the RSGFP signal was detected by fluorescence microscopy, cells were fixed by methanol. Afterward, chlamydial inclusions were stained by fluorescein isothiocyanate (FITC)-labeled monoclonal chlamydial-LPS antibodies. In the counting of RSGFP-expressing C. pneumoniae inclusions and immunofluorescence-staining inclusions, 24 independent images were used. The counted IFU were used for calculating the ratio of the RSGFP-expressing C. pneumoniae inclusions and immunofluorescence-staining inclusions.

### Fluorescence microscopy.

A Keyence BZ-9000 fluorescence microscope (Keyence, Osaka, Japan) was used to detect a fluorescence signal of RSGFP-expressing chlamydiae in HEp-2 cells. In addition, untransformed or pRSGFPCAT-Cpn-transformed C. pneumoniae LPCoLN- or CV-6*-*infected cells were analyzed by immunofluorescence staining with mouse anti-chlamydial LPS antibody (green), which stained chlamydial inclusions. Evans blue was used for counterstaining of host cells (red).

### Transmission electron microscopy.

Untransformed or pRSGFPCAT-Cpn-transformed C. pneumoniae*-*infected cells were fixed with 2% paraformaldehyde and 2.5% glutaraldehyde in 0.1 M cacodylate buffer for 1 h. Postfixation was performed with 1% OsO_4_ in 0.1 M cacodylate buffer for 2 h. Samples were dehydrated with graded ethanol series and embedded in araldite (Fluka, Buchs, Switzerland). Ultrathin sections were stained with uranyl acetate and lead citrate and were examined with a JEOL 1011 transmission electron microscope (TEM) (JEOL, Tokyo, Japan).

### Whole-genome and plasmid sequence.

*Chlamydia* spp. were cultivated on HEp-2 cells and purified by centrifugation. Genome DNA of *Chlamydia* spp. was extracted using the Nucleo Spin tissue kit (Macherey-Nagel, Dueren, Germany) and proteinase K digestion. From extracted genomic DNA, we prepared sequencing libraries using either the Illumina Nextera XT kit according to the manufacturer’s instructions or the Illumina Nextera kit according to a recently published protocol (41). Libraries were sequenced on the Illumina NextSeq 500 instrument in a 2- by 151-bp paired-end run (Illumina, San Diego, CA, USA).

For all the libraries, sequence contamination was removed with Deconseq v.0.4.3 (42) and the human reference genome GRCh38.p12. Quality control checks on raw sequence data were done with FastQC v.0.11.5 (43), and then data were trimmed and filtered with Trimmomatic (44) applying minimum quality 28 (parameters, SLIDINGWINDOW:3:28 MINLEN:50).

For the read-based analysis, sequence reads were mapped with bwa v.0.7.16a (45) to their reference genomes; more specifically, C. felis N.I. was mapped to its reference C. felis Fe/C-56 DNA (GenBank accession number AP006861) and library C. pneumoniae CV-6 was mapped to its reference C. pneumoniae CV-14 (GenBank accession number LN846996.1). In addition, the sequence reads were mapped to the sequence of the shuttle vector pRSGFPCAT-Cpn in order to ensure their presence or absence and the possible variants.

The alignments were coverted to bam format, sorted, and indexed with SAMtools (46). Afterward, duplicates were removed with Picard tools v.2.14.0 (https://broadinstitute.github.io/picard/). The result output was submitted for variant calling with freebayes v.1.1.0-54 (47). The vcf files were filtered by removing any sites with an estimated probability of not being polymorphic of less than phred 20. Mapping statistics of the bam files were obtained with bbmap v.37.61 (https://sourceforge.net/projects/bbmap/). The variant calling files were summarized and inspected with bcftools v.16 (46).

In order to compare the library C. pneumoniae CV-6-pRSGFPCAT-Cpn to library C. pneumoniae CV-6, a *de novo* assembly of the library C. pneumoniae CV-6 was performed with SPADES v.3.11.1 (48) with the careful parameter and a contig minimum of 500. The resulting contigs were filtered by length, discarding those shorter than 500 bp. The resulting set of contigs was evaluated with Quast v.4.6.3 (49) and used as the reference for the library C. pneumoniae CV-6-pRSGFPCAT-Cpn, repeating the read-based analysis.

Reads that were not mapping to their references were extracted with SAMtools and aligned to the nt NCBI database using blastn v.2.6.0 (50). The resulting output was then classified taxonomically with MEGAN v.6.11.1 (51) with a specified weighted last common ancestor (LCA) algorithm, a minimum score of 50, and a Max Expected of 0.1 as parameters and the nucleotide accession to NCBI taxonomy (nucl_acc2tax-Mar2018) as mapping file. Inspection of these classified unmapped reads revealed no chlamydial DNA sequence.

### Plasmid.

Plasmid maps were made by SnapGene. Using CLUSTALW in GenomeNet (http://www.genome.jp/), sequence data were compared to reference sequences (pCpnE1 and pRSGFPCAT).

### PCR.

PCR was performed to check whether *Chlamydia* spp. harbored the plasmid. Isolated *Chlamydia* spp. were boiled in TE buffer (10 mM Tris-HCl, pH 8.0, 1 mM EDTA) for 10 min, and 5 µl was used as a PCR template. The total 50-µl reaction mixture includes 20 mM deoxynucleoside triphosphate (dNTP) mix, 50 mM MgCl_2_, 20 µM primers, and 2.5 U *Taq* polymerase (Invitrogen, Karlsruhe, Germany). PCR primers and conditions are listed in Table S6 in the supplemental material (18, 52–55). The amplified products were electrophoresed on a 3% agarose gel.

10.1128/mSphere.00412-18.10 TABLE S6 Primers and PCR conditions used in this study. Download Table S6, DOCX file, 0.02 MB. Copyright © 2018 Shima et al. 2018 Shima et al. This content is distributed under the terms of the Creative Commons Attribution 4.0 International license.

### Statistics.

Data are indicated as mean ± standard error of the mean (SEM). Statistical analysis was performed with GraphPad Prism 7 statistical software. Sidak’s multiple comparison was used in the case that one-way analysis of variance showed statistically significant values (*P* values of ≤0.05). In Sidak’s multiple comparison, *P* values of ≤0.05 were considered statistically significant.

### Accession number(s).

All raw sequence data sets were deposited at the National Center for Biotechnology (NCBI) Sequence Read Archive (SRA) under Bioproject PRJNA473125 with SRA accession number SRP148963. Other accession numbers were SRR7217469 (C. felis N.I.), SRR7217470 (C. pneumoniae CV-6), and SRR7217471 (C. pneumoniae CV-6*-*pRSGFPCAT-Cpn).
