# Supplementary figures and images for: The Genetic Transformation of Chlamydia pneumoniae
Source: mSphere. 2018 Oct 10;3(5):e00412-18. doi: 10.1128/mSphere.00412-18 (PMC6180227; doi:10.1128/mSphere.00412-18)

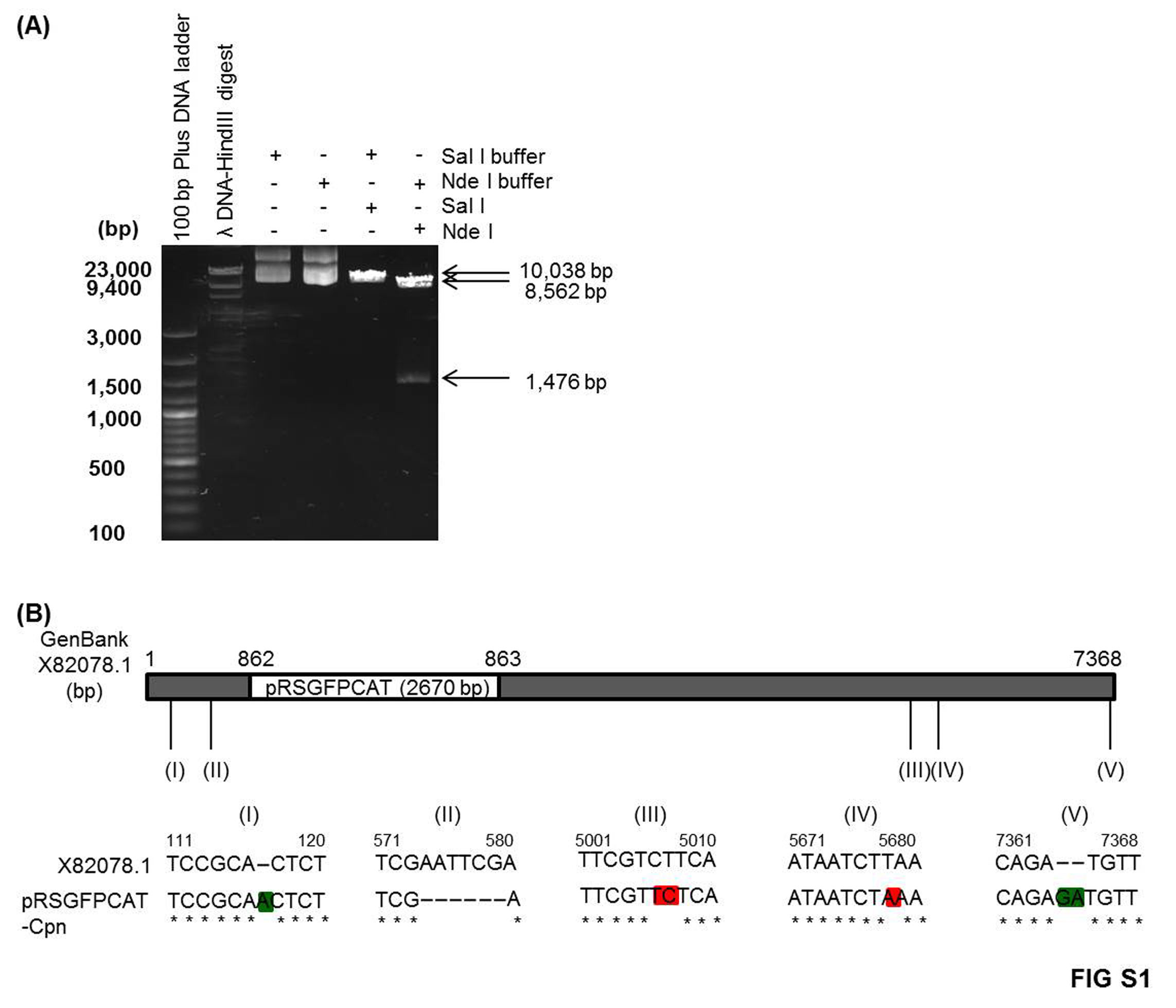

Supplement: FIG S1 [file sph005182657sf1.tif]

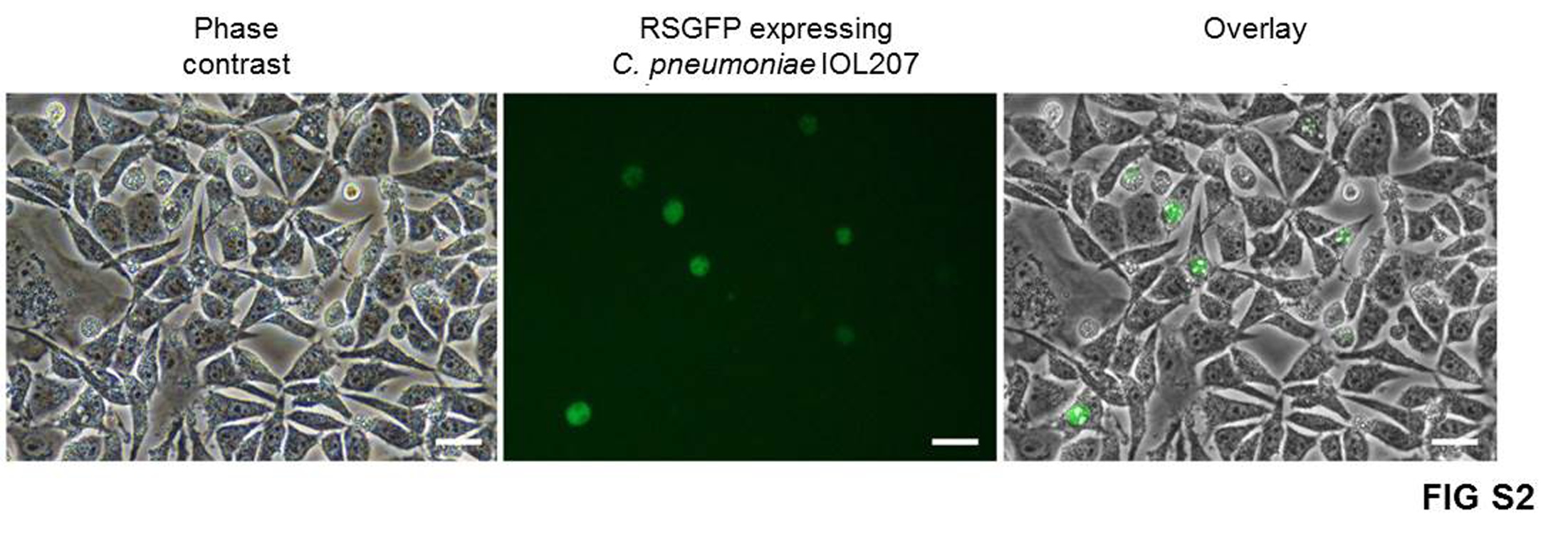

Supplement: FIG S2 [file sph005182657sf2.tif]

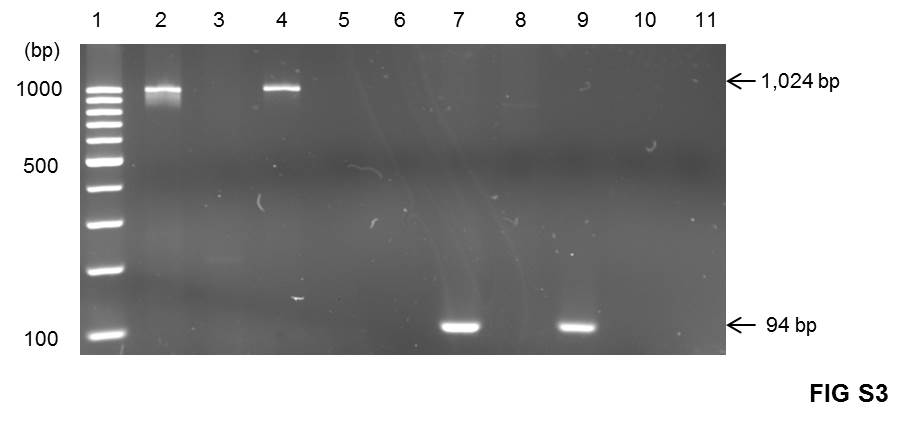

Supplement: FIG S3 [file sph005182657sf3.tif]

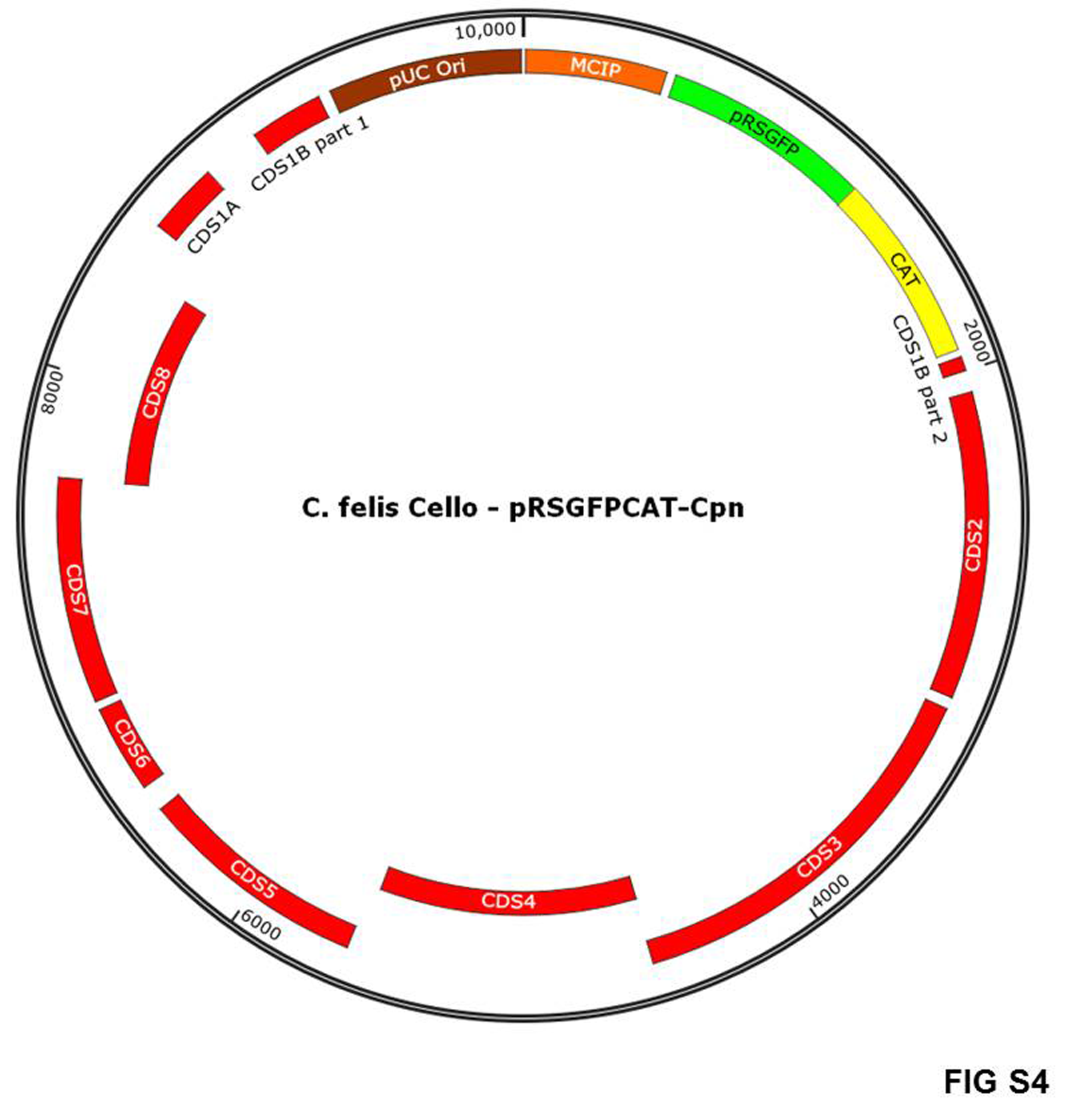

Supplement: FIG S4 [file sph005182657sf4.tif]
