# Supplementary material for: The Genetic Transformation of Chlamydia pneumoniae
Source: mSphere. 2018 Oct 10;3(5):e00412-18. doi: 10.1128/mSphere.00412-18 (PMC6180227; doi:10.1128/mSphere.00412-18)
Supplement: TABLE S1 [file sph005182657st1.docx]

| **Table S1** Sequence homology between *C. pneumoniae* N16 and other *Chlamydia* spp. | | | | | | | | | |  |  |
| --- | --- | --- | --- | --- | --- | --- | --- | --- | --- | --- | --- |
| Species | Strain | Plasmid | Total | CDS1 | CDS2 | CDS3 | CDS4 | CDS5 | CDS6 | CDS7 | CDS8 |
|  |  |  | CDSs |  |  |  |  |  |  |  |  |
| *C. pneumoniae* | N16 | pCpnEI | 100 | 100 | 100 | 100 | 100 | 100 | 100 | 100 | 100 |
| *C. pneumoniae* | LPCoLN | pCpnKo | 94 | 59 | 99 | 99 | 99 | 98 | 99 | 98 | 99 |
| *C. felis* | Fe/C-56 | pCfe1 | 65 | 47 | 67 | 68 | 77 | 64 | 76 | 70 | 62 |
| *C. felis* | N.I. | pCfelis | 65 | 47 | 67 | 68 | 77 | 64 | 76 | 70 | 62 |
| *C. pecorum* | L1 | CpecL1 | 65 | 47 | 65 | 69 | 76 | 65 | 75 | 64 | 64 |
| *C. caviae* | 03DC25 (GPIC) | pCpGP1 | 65 | 47 | 64 | 67 | 67 | 65 | 76 | 60 | 64 |
| *C. trachomatis* | L2 | pL2 | 60 | 47 | 61 | 59 | 66 | 60 | 68 | 50 | 57 |
| *C. muridarum* | MoPn/Nigg | pMoPn | 59 | 44 | 62 | 59 | 67 | 60 | 70 | 61 | 58 |
| BLAST-Needleman-Wunsch Global Align Nucleotide Sequence was used to compare sequence homology. | | | | | | | | | | | |
| Each sequence homology was calculated as a percentage compared to C. pneumoniae N16. N.I.; Not identified. | | | | | | | | | | | |
