# Supplementary material for: The Genetic Transformation of Chlamydia pneumoniae
Source: mSphere. 2018 Oct 10;3(5):e00412-18. doi: 10.1128/mSphere.00412-18 (PMC6180227; doi:10.1128/mSphere.00412-18)
Supplement: TABLE S2 [file sph005182657st2.docx]

| **Table S2** Whole genome sequence comparison  between *C. pneumoniae* CV-14 and *C. pneumoniae* CV-6 | |
| --- | --- |
|  | *C. pneumoniae* CV-6 |
| % Mapped reads | 99.92 |
| % Unmapped reads | 0.08 |
| % Reference bases covered | 99.9993 |
| Single nucleotide polymorphisms (SNPs) | 8 |
| Multi nucleotide polymorphisms (MNPs) | 0 |
| Indels < 5 bp | 2 |
| Inversions | 0 |
| % identity | 99.99829007 |
