# Supplementary material for: The Genetic Transformation of Chlamydia pneumoniae
Source: mSphere. 2018 Oct 10;3(5):e00412-18. doi: 10.1128/mSphere.00412-18 (PMC6180227; doi:10.1128/mSphere.00412-18)
Supplement: TABLE S3 [file sph005182657st3.docx]

| **Table S3** Whole genome sequence comparison  between wild type *C. peumoniae* CV-6 and *C. peumoniae* CV-6-pRSGFPCAT-Cpn | |
| --- | --- |
|  | *C. pneumoniae* CV-6-pRSGFPCAT-Cpn |
| % Mapped reads | 99.89 |
| % Unmapped reads | 0.11 |
| % Reference bases covered | 99.9998 |
| Single nucleotide polymorphisms (SNPs) | 9 |
| Multi nucleotide polymorphisms (MNPs) | 0 |
| Indels < 5 bp | 0 |
| Inversions | 0 |
| % identity | 99.99902058 |
| Reads of *C. pneumoniae* CV-6-pRSGFPCAT-Cpn was compared to wild type *C. peumoniae* CV-6 . | |
