# Supplementary material for: The Genetic Transformation of Chlamydia pneumoniae
Source: mSphere. 2018 Oct 10;3(5):e00412-18. doi: 10.1128/mSphere.00412-18 (PMC6180227; doi:10.1128/mSphere.00412-18)
Supplement: TABLE S4 [file sph005182657st4.docx]

| **Table S4** Whole genome sequence comparison  between *C. felis* Fe/C-56 and *C. felis* N.I. | |
| --- | --- |
|  | *C. felis* N.I. |
| % Mapped reads | 99.97 |
| % Unmapped reads | 0.03 |
| % Reference bases covered | 99.9956 |
| Single nucleotide polymorphisms (SNPs) | 93 |
| Multi nucleotide polymorphisms (MNPs) | 0 |
| Indels >= 5 bp | 1 |
| Indels <= 5 bp | 10 |
| Inversions | 0 |
| % identity | 99.98533748 |
| Reads of *C. felis* N.I. was compared to *C. felis* Fe/C-56. | |
