# Supplementary material for: The Genetic Transformation of Chlamydia pneumoniae
Source: mSphere. 2018 Oct 10;3(5):e00412-18. doi: 10.1128/mSphere.00412-18 (PMC6180227; doi:10.1128/mSphere.00412-18)
Supplement: TABLE S5 [file sph005182657st5.docx]

| **Table S5** Sequence homology among *C. felis* N.I., *C. trachomatis* and *C. pneumoniae*. | | | | | | | | | | |  |
| --- | --- | --- | --- | --- | --- | --- | --- | --- | --- | --- | --- |
| Species | Strain | Plasmid | Total | CDS1 | CDS2 | CDS3 | CDS4 | CDS5 | CDS6 | CDS7 | CDS8 |
|  |  |  | CDSs |  |  |  |  |  |  |  |  |
| *C. felis* | N.I. | pCfelis | 100 | 100 | 100 | 100 | 100 | 100 | 100 | 100 | 100 |
| *C. trachomatis* | L2 | pL2 | 62 | 62 | 61 | 59 | 66 | 65 | 72 | 60 | 62 |
| *C. pneumoniae* | LPCoLN | pCpnKo | 68 | 71 | 64 | 67 | 77 | 64 | 77 | 71 | 62 |
| *C. pneumoniae* | N16 | pCpnEI | 65 | 47 | 67 | 68 | 77 | 64 | 76 | 70 | 62 |
| BLAST-Needleman-Wunsch Global Align Nucleotide Sequence was used to compare sequence homology. | | | | | | | | | | | |
| Each sequence homology was calculated as a percentage compared to *C. felis* N.I. N.I.; Not identified. | | | | | | | | | | | |
