# Supplementary material for: The Genetic Transformation of Chlamydia pneumoniae
Source: mSphere. 2018 Oct 10;3(5):e00412-18. doi: 10.1128/mSphere.00412-18 (PMC6180227; doi:10.1128/mSphere.00412-18)
Supplement: TABLE S6 [file sph005182657st6.docx]

| **Table S6** Primers and PCR conditions used in this study. | | |  |  |  |  |  |  |  |  |
| --- | --- | --- | --- | --- | --- | --- | --- | --- | --- | --- |
| Primer | Sequence (5´- 3´) | Target | First |  | PCR condition |  | Final delay | Cycle | Amplicon (bp) | Reference |
|  |  |  | denaturing | denaturing | Annealing | Extension |  |  |  |  |
| N16 pgp F | ATGGGATCTCAGCAGATTGT | *C. pneumoniae* plasmid | 94°C 5min | 94°C 30s | 47°C 1 min | 72°C 1 min | 72°C 7 min | 30 | 198 | N.A.* |
| N16 pgp R | GGCTGTTGCTTGATTGATTA |  |  |  |  |  |  |  |  |  |
|  |  |  |  |  |  |  |  |  |  |  |
| pCF01F | GGCAACTTTATCTCCAATCACC | *C. felis* plasmid | 95°C 5min | 95°C 30s | 54°C 1 min | 72°C 1 min | 72°C 7 min | 30 | 94 | [53] |
| pCF01R | CTTTCCAGCTTCATAGAACCATC |  |  |  |  |  |  |  |  |  |
|  |  |  |  |  |  |  |  |  |  |  |
| pCFelis-F | CACACTAGGGAGACAATTTCCA | *C. felis* plasmid | 95°C 5min | 95°C 30s | 54°C 1 min | 72°C 1 min | 72°C 7 min | 30 | 1024 | [18] |
| pCFelis-R | GACCACTATCCCTGAGATCCGA |  |  |  |  |  |  |  |  |  |
|  |  |  |  |  |  |  |  |  |  |  |
| C.peco-P-F | GTTCACACTCTGCCTCATC | *C. pecorum* plasmid | 95°C 5min | 95°C 30s | 47°C 1 min | 72°C 1 min | 72°C 7 min | 30 | 522 | [52] |
| C.peco-P-R | CCTATTTATTGGCGTCTAGG |  |  |  |  |  |  |  |  |  |
|  |  |  |  |  |  |  |  |  |  |  |
| Ct pgp-F | TCAAGGACCAGCAAATAATC | *C. trachomatis* plasmid | 94°C 5min | 94°C 30s | 47°C 1 min | 72°C 1 min | 72°C 7 min | 30 | 320 | N.A. |
| Ct pgp-R | GAATAACCCGTTGCATTGAA |  |  |  |  |  |  |  |  |  |
|  |  |  |  |  |  |  |  |  |  |  |
| C.cavi-P-F | CAGGTCTTGCAGCGACAACA | *C. caviae* plasmid | 95°C 5min | 95°C 30s | 47°C 1 min | 72°C 1 min | 72°C 7 min | 30 | 287 | [54] |
| C.cavi-P-R | ACGTTCACCGTTCACGCTTA |  |  |  |  |  |  |  |  |  |
|  |  |  |  |  |  |  |  |  |  |  |
| C.muri-P-F | TGTCACAGCGGTTGCTCTAA | *C. muridarum* plasmid | 95°C 5min | 95°C 30s | 47°C 1 min | 72°C 1 min | 72°C 7 min | 30 | 317 | [55] |
| C. muri-P-R | CTATGCTGCAAGGAGGTAAG |  |  |  |  |  |  |  |  |  |
| * N.A., Not applicable | |  |  |  |  |  |  |  |  |  |
